# Breast metastasis from medullary thyroid carcinoma: a report of a case

**DOI:** 10.1186/s40792-021-01273-w

**Published:** 2021-08-19

**Authors:** Yoko Omi, Hidenori Kamio, Yusaku Yoshida, Kenta Masui, Tomoko Yamamoto, Yoji Nagashima, Takahiro Okamoto

**Affiliations:** 1 Department of Breast and Endocrine Surgery, Tokyo Women’s Medical University, 8-1 Kawada-cho, Shinjuku-ku, Tokyo, Japan; 2 Department of Diagnostic Pathology, Tokyo Women’s Medical University, 8-1 Kawada-cho, Shinjuku-ku, Tokyo, Japan

**Keywords:** Medullary thyroid carcinoma, Calcitonin, Washout, Immunohistochemistry

## Abstract

**Background:**

Metastasis to the breast is rare. We herein report a patient with metastatic medullary thyroid carcinoma to the breast for whom measuring the calcitonin level was an important clue to the correct diagnosis.

**Case presentation:**

A 54-year-old woman visited our hospital for the treatment of recurrent metastatic medullary thyroid carcinoma due to multiple endocrine neoplasia 2A and breast cancer. Positron emission tomography performed before the operation for metastatic medullary thyroid carcinoma recurrence in the neck showed the accumulation of ^18^F-fluorodeoxyglucose in the bilateral breast at sites other than the disease in the neck. Ultrasonography revealed multiple tumors in both breasts. A core needle biopsy of three breast tumors was performed. Microscopically, the tumor cells showed solid growth and did not show a tubular structure. She was diagnosed with triple-negative invasive ductal carcinoma. Post-operative positron emission tomography was performed as the serum calcitonin level increased after the operation. The accumulation of ^18^F-fluorodeoxyglucose in the bilateral breast tumors and lymph nodes in the neck was noted. The possibility of the breast tumors being metastasis of metastatic medullary thyroid carcinoma was considered. Needle aspiration was performed for three breast tumors. The calcitonin level of the washout fluid was measured and found to be ≥ 17,500 pg/mL. Immunohistochemistry showed that the tumor cells were calcitonin-positive and gross cystic disease fluid protein-15-negative. Vandetanib was started as recurrent metastatic medullary thyroid carcinoma with breast metastasis was finally diagnosed. The serum calcitonin level decreased after 1 month.

**Conclusion:**

Although breast metastasis of medullary thyroid carcinoma is rare, a correct diagnosis is indispensable for appropriate treatment. When a breast tumor shows atypical morphological features for breast cancer according to the histopathology in a patient with a history of cancer, metastasis to the breast should be considered. Calcitonin measurement of the needle washout fluid was useful for confirming metastatic medullary thyroid carcinoma.

## Background

Mammary malignant tumor originating from sites other than the breast accounts for < 2% of breast malignancies [1]. The therapy differs completely depending on its origin, so to avoid unnecessary intervention, making a differential diagnosis before starting the treatment is important.

We herein report a case of metastatic breast tumor from medullary thyroid carcinoma (MTC) diagnosed before the treatment had started.

## Case presentation

A 54-year-old woman visited our hospital for the treatment of recurrent MTC and breast cancer.

**Fig. 1:**
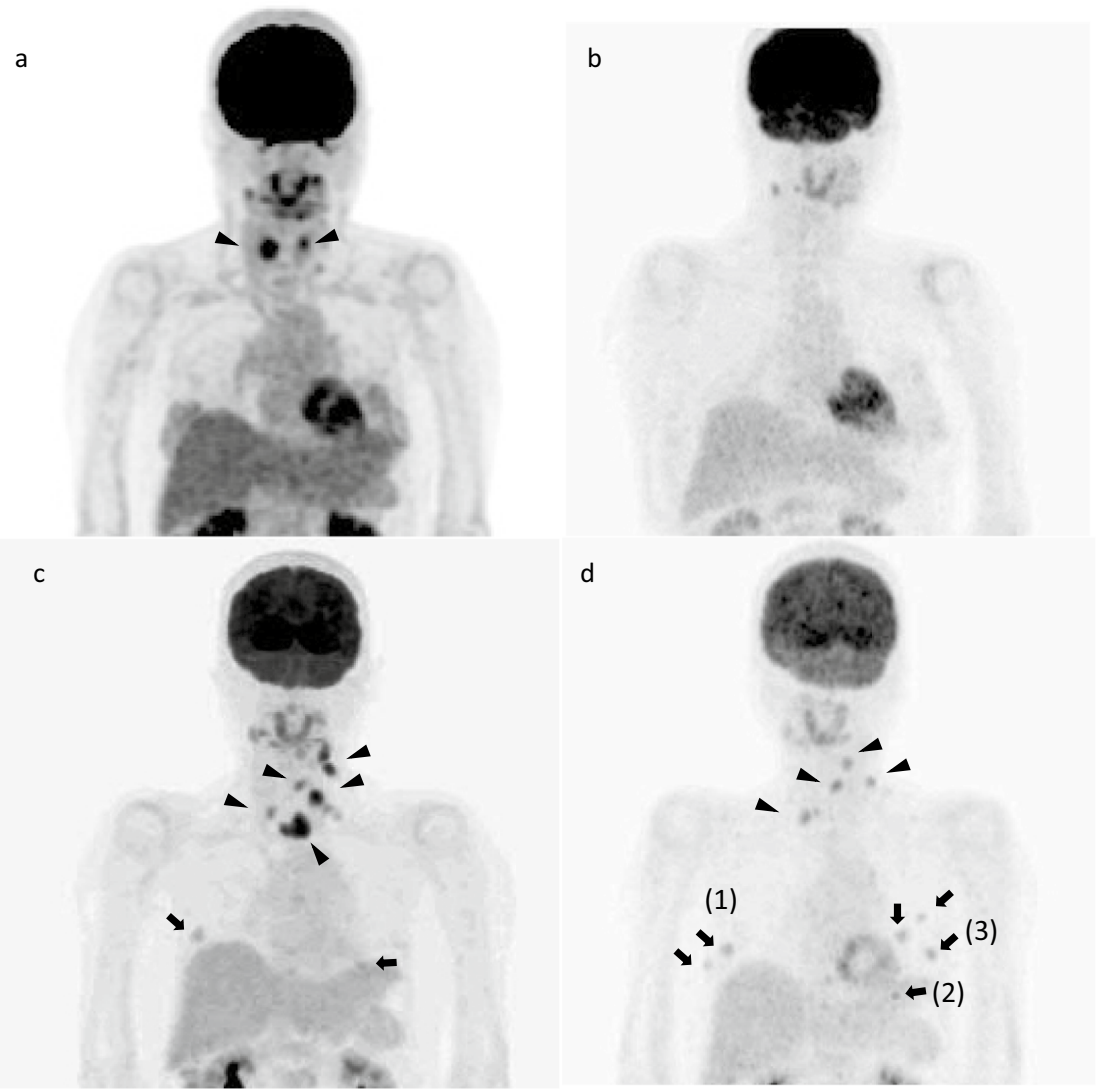
FDG PET–CT findings. **a** PET scan performed before the first surgery. FDG accumulation in the MTC in the thyroid (arrowheads). **b** PET scan performed after the first surgery. No abnormal accumulation was found in the neck or the breast. **c** PET scan performed before the second surgery. FDG accumulation was found in the neck, mediastinum (arrow heads), and bilateral breasts (arrows). **d** PET scan performed after the second surgery. FDG accumulation was found in the neck (arrow heads) and breast tumors (arrows). The numbers (1) to (3) correspond to the breast tumors in the text

**Fig. 2:**
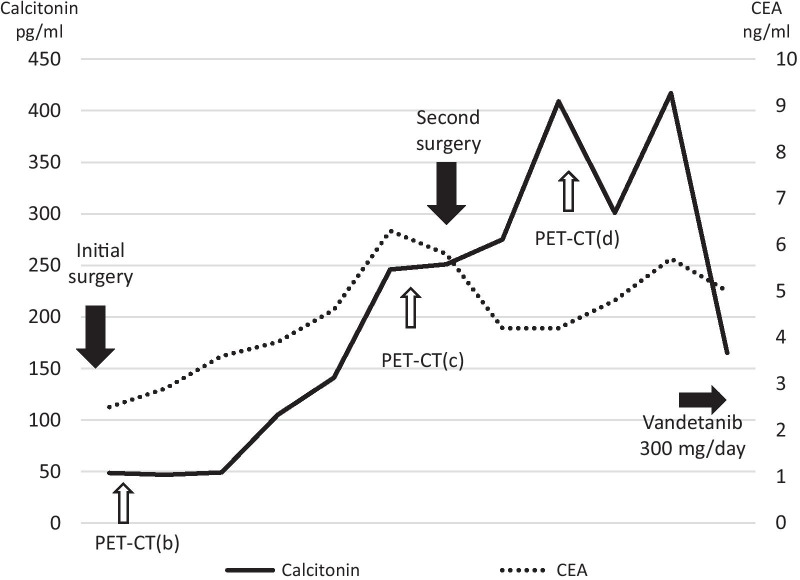
Blood test results and progress of treatment. Serum calcitonin level increased after the second surgery. Serum CEA level stayed as high as around upper limit of normal range. PET–CT (b to d) correspond to Fig. 1

**Fig. 3:**
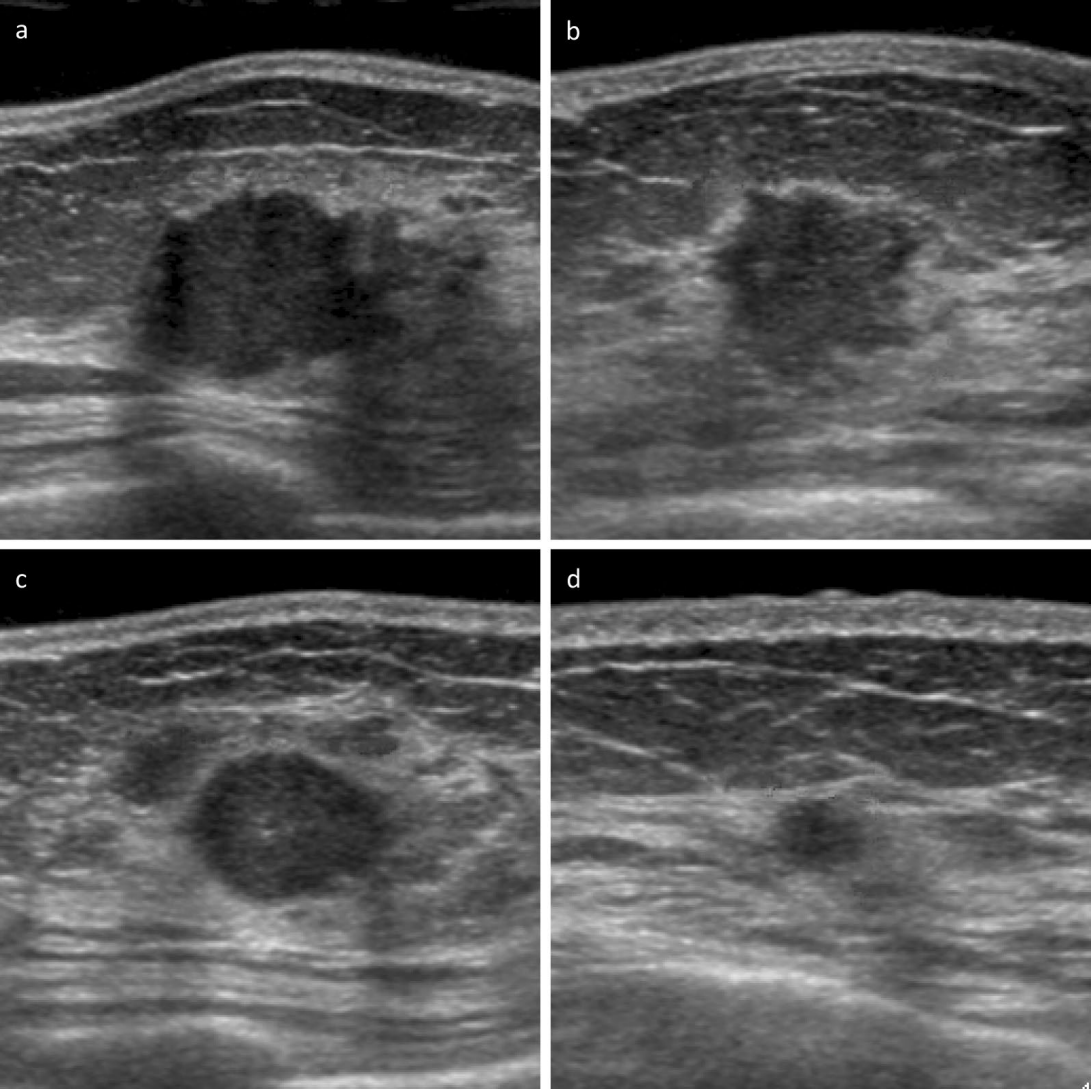
Ultrasonographical findings of the breast tumors. Multiple low echoic tumors with irregular margins in both breasts were observed. **a** The breast tumor in the right upper-outer area (1). **b** The breast tumor in the left lower-inner area (2). **c** The breast tumor in the left upper-outer area (3). **d** The breast tumor in the left upper-inner area

**Fig. 4:**
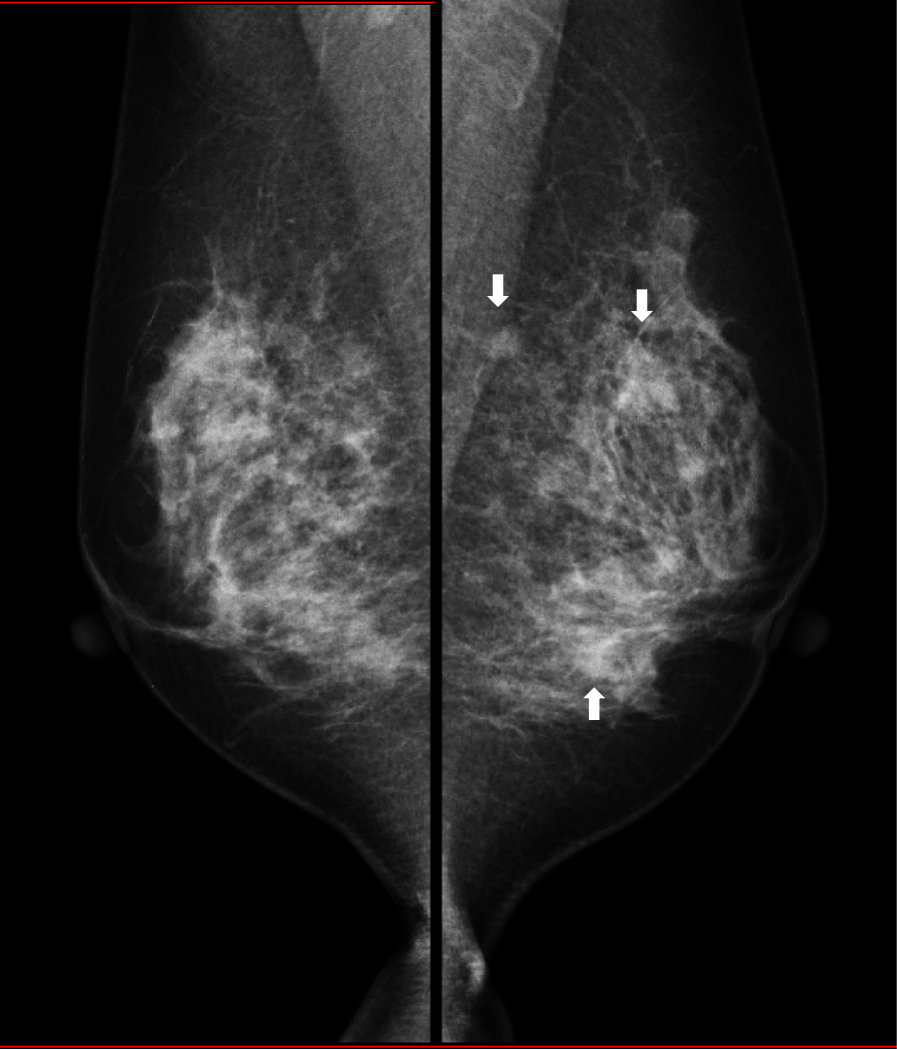
Mammographic findings. Focal asymmetric density is detected in the left breast (arrows)

She had undergone total thyroidectomy and neck lymph node dissection 3 years ago for MTC due to multiple endocrine neoplasia (MEN) 2A at another hospital. Her mother and brother had MTC. Genetic testing confirmed a c.1858 T > C pathogenic mutation in exon 10 at codon 620 of the RET gene. Positron emission tomography (PET) performed after the initial surgery showed that MTC in the thyroid was successfully removed no abnormal accumulation was found in the neck or the breast (Fig. 1a, b). After 2 years from the first operation, serum calcitonin level started to rise. After 2.5 years from the initial surgery, serum carcinoembryonic antigen (CEA) (normal range: ≤ 5 ng/mL) and calcitonin (normal range: ≤ 6.4 pg/mL) level reached to 6.3 ng/mL and 246 pg/mL, respectively (Fig. 2). PET searching for recurrent disease revealed the accumulation of ^18^F-fluorodeoxyglucose (FDG) in the neck, mediastinum, and bilateral breasts (Fig. 1c). Ultrasonography of the breast revealed multiple bilateral tumors 4–19 mm in size without axillary lymph node involvement (Fig. 3a–d). A mammogram showed asymmetric focal densities in the left breast (Fig. 4).

**Fig. 5:**
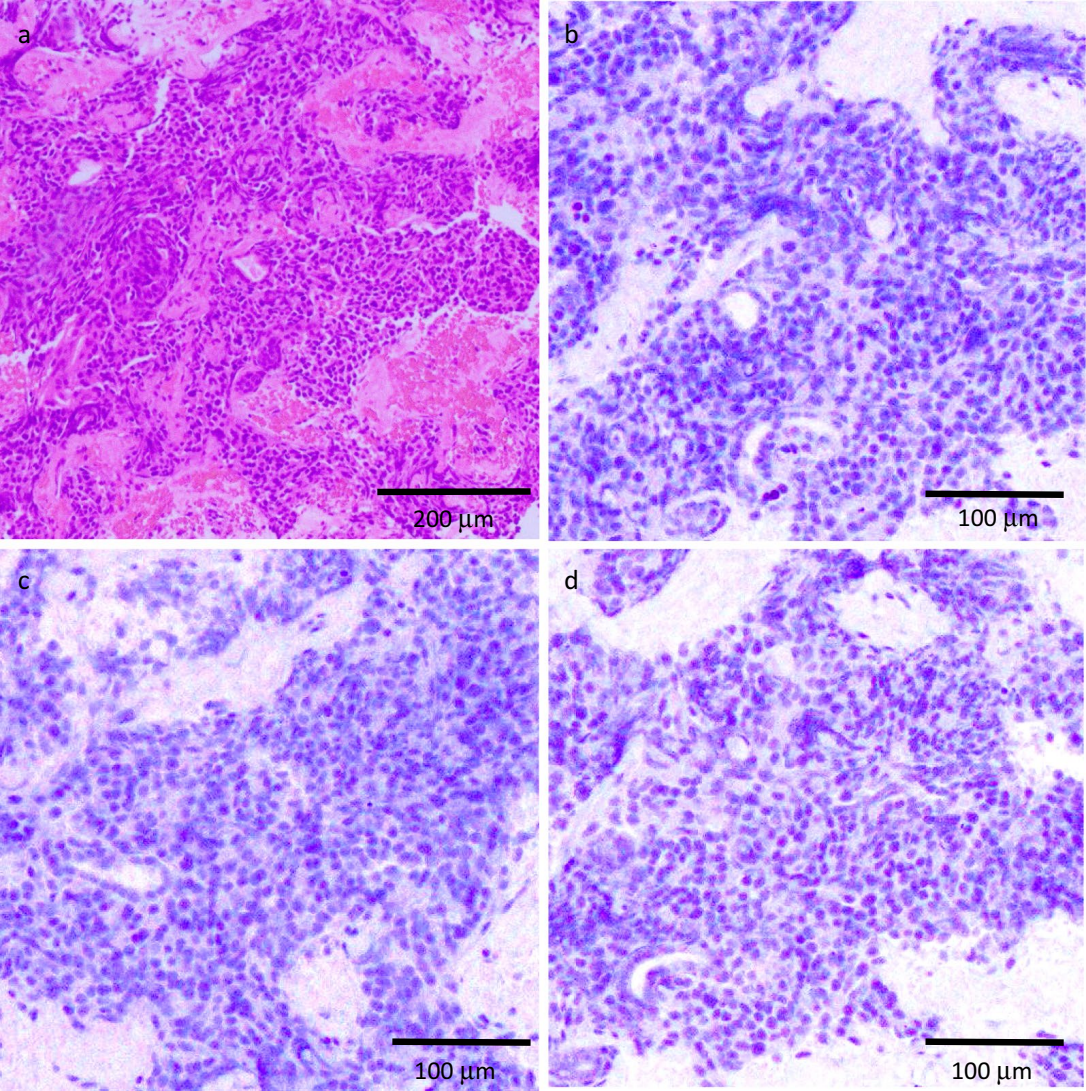
Histological findings of the breast tumor (1). **a** The tumor cells showed solid growth and tubular structure was not observed. (hematoxylin and eosin stain). **b** Estrogen receptor was negative. **c** Progesterone receptor was negative. **d** HER2 was negative

Core needle biopsy specimens of three tumors from different areas showed the same histopathological findings comprising solid growth and tubular structures diagnosed as invasive ductal carcinoma (Fig. 5a). Estrogen receptor, progesterone receptor, and human epidermal growth factor receptor (HER2) were all negative (Fig. 5b–d).

She was scheduled to receive neck surgery for the recurrent MTC, followed by neoadjuvant chemotherapy for the "triple-negative" breast tumors. Metastatic lymph nodes in the superior mediastinum and the left lateral neck region were removed. However, the neck surgery failed to provide sufficient palliation, as the post-operative serum calcitonin level increased to 409 pg/mL (Fig. 2). PET scan performed after second surgery showed the accumulation of FDG in the bilateral breast tumors and metastatic lymph nodes in the neck (Fig. 1d). She was therefore referred to our hospital for the further management of both neck and breast lesions.

**Fig. 6:**
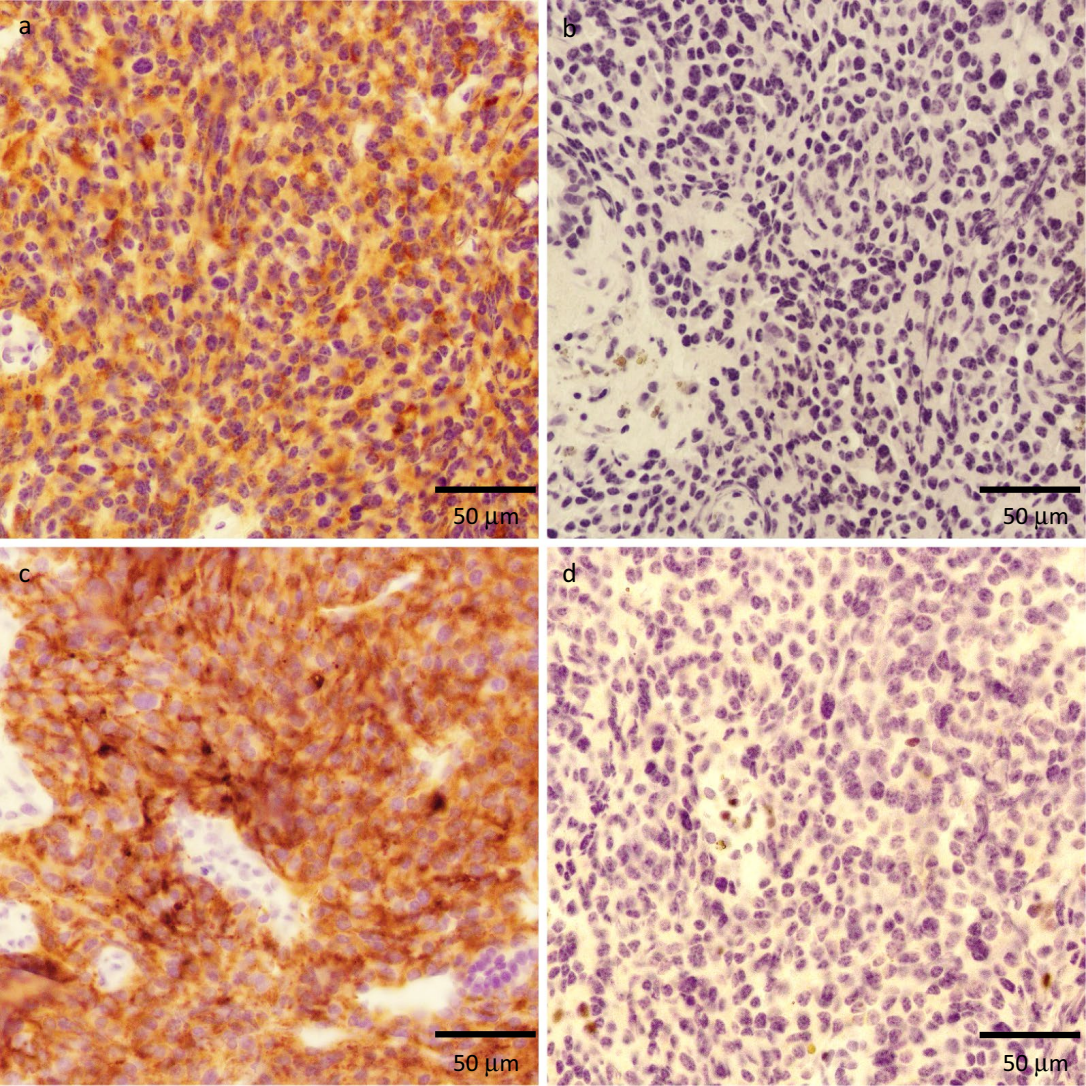
Immunohistochemical findings of the breast tumors. **a** Calcitonin was positive in the tumor (1). **b** GCDFP-15 was negative in the tumor (1). **c** CEA was positive in the tumor (2). **d** GATA3 was negative in the tumor (1)

Possibility of metastatic MTC was discussed as the breast tumors were multiple and bilateral, and their ultrasonographic features resembled each other. Fine-needle aspiration was performed to rule out the metastatic MTC before starting chemotherapy for the breast cancer. The calcitonin levels of diluted specimens obtained by adding 1 mL saline to fine-needle aspiration from the 3 corresponding breast tumors were 17,500 pg/mL, 24,800 pg/mL, and 29,200 pg/mL. These extremely high values indicated that the breast tumors were metastatic lesions from MTC. An immunohistochemical examination of the core-biopsied materials was performed to ensure the result of fine-needle aspiration. Calcitonin staining was positive (Fig. 6a), whereas GCDFP-15 staining was negative in all three tumors (Fig. 6b). CEA was positive in breast tumor (2) (Fig. 6c) and negative in breast tumors (1) and (3). GATA3 was examined in the breast tumor (1) and was not found to be expressed (Fig. 6d). The patient has started taking vandetanib 300 mg per day following the final diagnosis of recurrent and metastatic breast lesions from MTC. The serum calcitonin level dropped down from 417 to 165 pg/mL after taking vandetanib for 1 month (Fig. 2).

## Discussion

Metastatic breast tumor from an extramammary malignancy is reported to occur in 0.3–2% of breast malignancies [1–3]. The lung, stomach, skin, and ovary are the most frequent original cancer sites [1–6]. The clinical features of the metastatic tumors to the breast often include superficial, well-defined, or slightly irregular margins and a location in the upper-outer area [3, 5, 7]. The existence of multiple or bilateral diseases as a characteristic of metastatic breast tumors is controversial [2, 3, 5–7]. A history of cancer or metastasis other than to the breast is also a clue to the diagnosis.

The thyroid is not an ordinary primary site, although some cases have been reported. Mandanas et al. reviewed 20 cases of MTC metastasis to the breast, including 5 MEN 2 patients in the literature [8]. Six patients had bilateral lesions, and the other four showed multiple tumors of the unilateral breast. Eighteen patients had distant metastases in other sites at the time of the diagnosis of breast involvement. Sixteen patients were successfully diagnosed by fine-needle aspiration cytology or a core needle biopsy, some of them with immunohistochemistry.

However, making a histopathological diagnosis of metastatic breast tumor can be challenging. Some types of cancer have a specific histological feature that can identify the origin. The presence of elastosis, carcinoma in situ, and calcification are common features of primary mammary carcinomas but rare in extramammary tumors [2]. It is essential to narrow down the diagnosis morphologically using hematoxylin–eosin-stained sections. Solid growth without tubular formation prompted us to suspect MTC metastasis in this case. Immunohistochemistry provides additional information concerning the differential diagnosis. Immunophenotyping using cytokeratin 7 and cytokeratin 20 is helpful [2]. Breast cancer is typically cytokeratin 7-positive and cytokeratin 20-negative [2]. The estrogen receptor is expressed in 80% of breast cancer cases, the progesterone receptor in 60%, and GCDFP-15 in 70% [2]. GATA3 is a zinc finger transcription factor with a higher sensitivity for triple-negative cancer than GCDFP-15, but it is expressed in urothelial carcinoma as well [9]. Since CEA and calcitonin are helpful tumor markers of MTC, their elevated levels may be a clue to the correct diagnosis. In addition, measuring the calcitonin level of the washout fluid from a fine-needle aspiration biopsy is a useful examination, as it has 97.9% sensitivity for MTC, regardless of the cytologic findings [10]. It is crucial to make every effort to achieve the correct diagnosis, since the appropriate treatments for metastatic breast tumors from MTC are completely different from those for primary breast cancer.

## Conclusion

When a breast tumor shows atypical morphological features for breast cancer according to histopathology in a patient with a history of cancer, metastasis to the breast should be considered, even though it is infrequent. The calcitonin level of the needle washout fluid was useful for confirming metastatic MTC in addition to immunostaining of CEA and calcitonin.

## Data Availability

All data generated or analyzed during this study are included in this published article.

## Abbreviations

MTC: Medullary thyroid carcinoma
MEN: Multiple endocrine neoplasia
PET: Positron emission tomography
FDG: ^18^F-fluorodeoxyglucose
HER2: Human epidermal growth factor receptor
GCDFP-15: Gross cystic disease fluid protein-15
CEA: Carcinoembryonic antigen
